# The Beauty is a beast: Does leachate from the invasive terrestrial plant *Impatiens glandulifera* affect aquatic food webs?

**DOI:** 10.1002/ece3.8781

**Published:** 2022-04-06

**Authors:** Jens G. P. Diller, Sophia Drescher, Mario Hofmann, Max Rabus, Heike Feldhaar, Christian Laforsch

**Affiliations:** ^1^ 26523 Animal Ecology I University of Bayreuth Bayreuth Germany; ^2^ 26523 Bayreuth Center for Ecology and Environmental Research (BayCEER) University of Bayreuth Bayreuth Germany

**Keywords:** *Acutodesmus obliquus*, allelochemicals, cross‐ecosystem effects, *Daphnia magna*, Invasive species, leachate

## Abstract

Invasive alien species are a major threat to ecosystems. Invasive terrestrial plants can produce allelochemicals which suppress native terrestrial biodiversity. However, it is not known if leached allelochemicals from invasive plants growing in riparian zones, such as *Impatiens glandulifera*, also affect freshwater ecosystems. We used mesocosms and laboratory experiments to test the impact of *I. glandulifera* on a simplified freshwater food web. Our mesocosm experiments show that leachate from *I. glandulifera* significantly reduced population growth rate of the water flea *Daphnia magna* and the green alga *Acutodesmus obliquus*, both keystone species of lakes and ponds. Laboratory experiments using the main allelochemical released by *I. glandulifera*, 2‐methoxy‐1,4‐naphthoquinone, revealed negative fitness effects in *D. magna* and *A. obliquus*. Our findings show that allelochemicals from *I. glandulifera* not only reduce biodiversity in terrestrial habitats but also pose a threat to freshwater ecosystems, highlighting the necessity to incorporate cross‐ecosystem effects in the risk assessment of invasive species.

## Graphical abstract

Invasive plant species are a major threat to ecosystems. We could show that allelochemicals from *I. glandulifera*, an invasive terrestrial plant species, not only reduce biodiversity in terrestrial habitats but also pose a threat to freshwater ecosystems, highlighting the necessity to incorporate cross‐ecosystem effects in the risk assessment of invasive species.

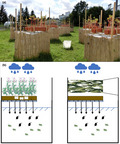

## 1 INTRODUCTION

Invasive species have direct effects on the biodiversity and stability of ecosystems, for instance when they outcompete the native fauna and flora (Beck et al., 2008). In terrestrial invasive plants, this may result in monospecific stands of the invasive species in the invaded habitats (Hierro & Callaway, 2003). The formation of such monospecific stands of invasive plants can be achieved by releasing so‐called allelochemicals into the environment, against which native plant species are often not adapted to (Bergstrom et al., 2009; Callaway & Ridenour, 2004; Lind & Parker, 2010; Van Kleunen et al., 2010). Allelochemicals are secondary metabolites produced by plants that are not needed for basic metabolism in the donor plant (Fraenkel, 1959), instead they benefit the donor plant as they act, for example, as defense against herbivores (Meiners et al., 2012). Further, they can be released by the donor plant to reduce growth and germination rates in neighboring plants, thus hampering their development (Yoneyama & Natsume, 2010). This is a common phenomenon in the system of the plant kingdom: For instance, common butterbur, *Petasites hybridus* (Asteraceae), produces pyrrolizidine alkaloids (PAs) (Kisielius et al., 2020), bracken ferns, *Pteridium* spp., produce carcinogenic illudane glycosides (Skrbic et al., 2021), and the black walnut tree, *Juglans nigra*, releases juglone (5‐hydroxy‐1,4‐naphthoquinone) (Achatz et al., 2014; Rietveld, 1983; Soderquist, 1973), which affect neighboring terrestrial plants. If donor plants grow in riparian areas, allelochemicals may cross ecosystem boundaries (Jackrel & Wootton, 2015) and leach into the adjacent water bodies, especially during precipitation, which was shown for allelochemicals from bracken (Skrbic et al., 2021). Ecotoxicological studies in the laboratory, performed with the extracted allelochemical juglone and other natural toxins, revealed negative effects of these compounds on water dwelling organisms, such as an increased mortality in zoo‐ and phytoplankton (Griffiths et al., 2021; Westfall et al., 1961; Wright et al., 2007). Based on these findings, invasive terrestrial plants, that form monospecific stands in riparian areas may not only affect terrestrial native plant species they may additionally affect the neighboring aquatic ecosystems. Griffith et al. (2021) could show that plant produced phytotoxic alkaloids, e.g., from the lupin (*Lupinus spp*.) or the ragwort (*Senecio jacobaea*), elicit effects on life history and morphological parameters in *Daphnia magna*. Another plant releasing allelochemicals is the Himalayan Balsam, *Impatiens glandulifera*, which is invasive in large parts of the northern hemisphere. It grows predominantly in moist areas, such as riverbanks and wetlands and often forms monospecific stands (*Impatiens glandulifera (IPAGL)[World distribution]| EPPO Global Database* (2019)). *I*. *glandulifera* prevails itself against competitors with the production of different allelochemicals, with 2‐methoxy‐1,4‐naphthoquinone (2‐MNQ) being the main compound (Lobstein et al., 2001). It has been shown that extracts from *I. glandulifera* and purified 2‐MNQ from *I. glandulifera* itself inhibit the germination and growth of native terrestrial plants such as the common nettle *Urtica dioica* or ectomycorrhizal fungi like *Pisolithus tinctorius* (Bieberich et al., 2018; Ruckli et al., 2014). Allelochemicals produced by *I. glandulifera* can leach from the plants’ leaves, shoots, and roots during precipitation with 2‐MNQ reaching concentrations of up to 12 mg/L in the surrounding soil (Lobstein et al., 2001; Ruckli et al., 2014). Therefore, due to its preference for riparian habitats, considerable quantities of allelochemicals can potentially leach from the monospecific stands into the adjacent water bodies. Thus, we anticipate that leachate from *I. glandulifera* including its secondary metabolite 2‐MNQ negatively affects freshwater dwelling organisms.

To test our hypothesis, we investigated the effects of natural leachate and purified 2‐MNQ, as the primary allelochemical released from *I. glandulifera*, on two freshwater model organisms: The green alga *Acutodesmus obliquus* as primary producer and the planktonic crustacean *D. magna* as primary consumer. To test for effects under semi‐natural conditions, we conducted a mesocosm study using both organisms, representing a simplified limnetic food web. In addition, we performed laboratory studies on both organisms with purified 2‐MNQ, as model allelochemical, to distinguish between direct (mortality, number of offspring, population growth) or indirect (negative effects on *D. magna* from direct effects on *A. obliquus*) effects from the leachate in the mesocosm study.

## 2 METHODS

### 2.1 Chemicals

Commercially available 2‐methoxy‐1,4‐naphthoquinone (2‐MNQ; MW = 188.19 g/mol; logKow = 1.13 Table S1) was purchased from Sigma‐Aldrich (CAS number: 2348–82–5; Darmstadt, Germany).

The solvent dimethyl sulfoxide (DMSO) was obtained from Bernd Kraft (Duisburg, Germany). The fluorescence dye 2’,7’‐dichlorofluorescin diacetate (DCFDA) was obtained from Sigma‐Aldrich (CAS number: 4091‐99‐0; Darmstadt, Germany).

### 2.2 Animal husbandry

We used 13 laboratory‐cultured clones of *D. magna (K34J)* for the experiments (Tab. S6), isolated from ponds near Munich, Germany. The animals were cultured in 1 L glass beakers (WECK GmbH, Wehr‐Oeflingen, Germany) in Elendt M4‐medium (Elendt, 1990) in a climate chamber at 20°C ± 0.5°C and a day and night rhythm of 14: 9 h, with a half hour each of dusk and dawn and fed daily ad libitum with the green algae *A. obliquus* (SAG‐Strain: 276‐3a, Culture Collection of Algae at Goettingen University, Goettingen). The M4 medium for *D. magna* was replaced weekly.

### 2.3 Algae culturing

An *A. obliquus* strain (SAG‐Strain. 276‐3a, Culture Collection of Algae at Goettingen University, Goettingen) was used. As growth medium for *A. obliquus*, we used Z/4 medium (Staub, 1961) with 21: 3 day/night cycle at 20°C. We cultured the algae under semi‐static conditions and measured the carbon content with a photometer (UviLine 9100, SI Analytics, Weinheim, Wavelength: 800 nm). The algae were harvested in the exponential growth phase.

### 2.4 Mesocosm design and experimental setup

**FIGURE 1:**
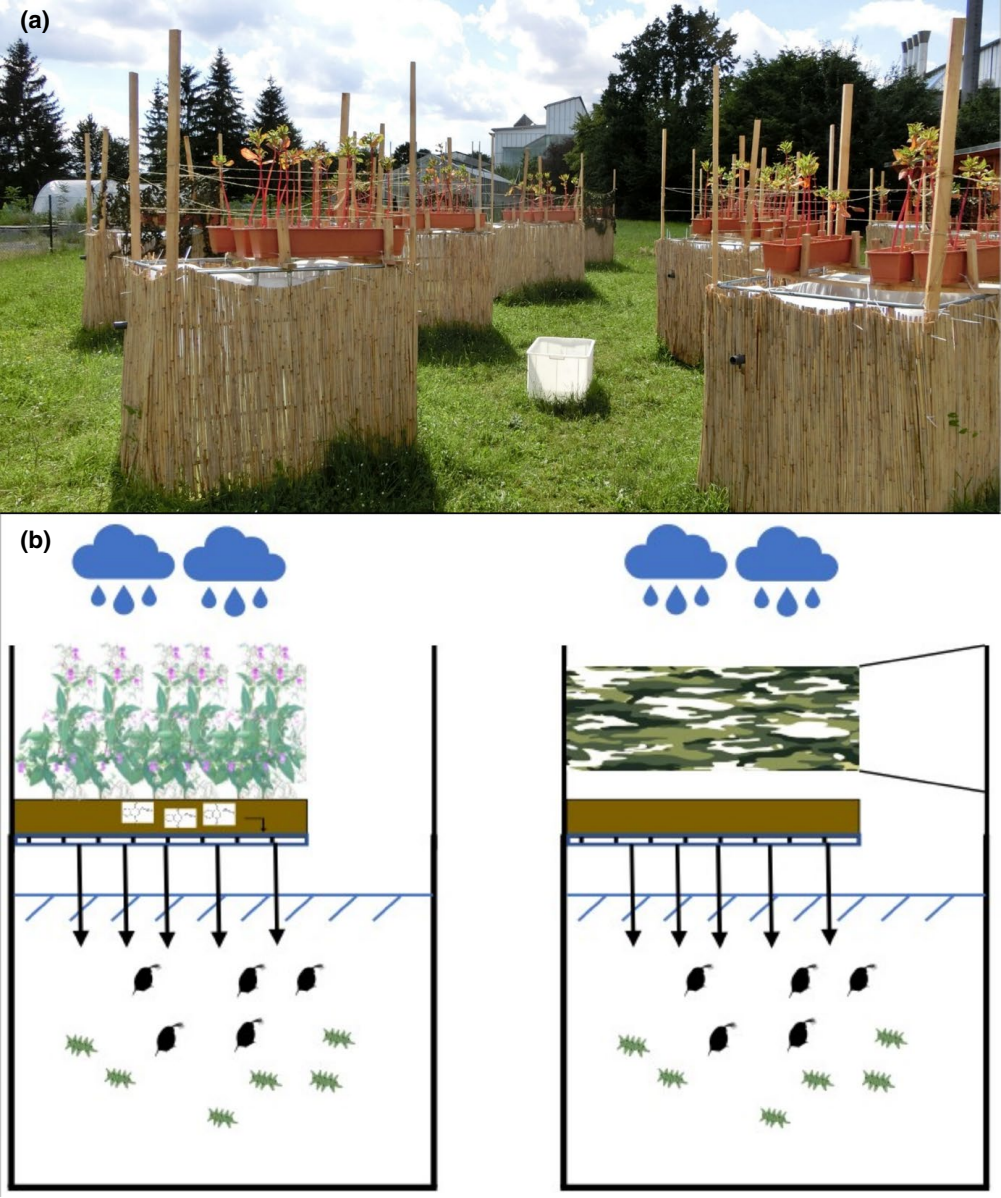
(a) The mesocosms for the experiment, with soil +*I. glandulifera* as treatment group and with only soil +camouflage net as control. (b) Schematic drawing of the mesocosms, on the left side with *I. glandulifera*, on the right side without the plant, but with a camouflage net to mimic shading by the plants

The experiments using 12 mesocosms per treatment were conducted in an outdoor experimental area at the University of Bayreuth, Germany (49°92´67”N; 11°58´28”E) (Figure 1a,b). The mesocosms were made of IBC Water tanks (Material: HDPE, volume 1000 L, length 120 cm, width 100 cm, height 116 cm; Auer Packaging GmbH, Amberg, Germany). The top was removed, and they were surrounded by 600 × 100 cm rush mats (Hornbach Holding AG & Co. KGaA) to shade them laterally as heat protection. The soil and plants were placed in 15 l plastic flowerpots (W × D × H: 80 × 17 × 14 cm; HORNBACH Holding AG & Co. KGaA), which were three times pierced at the bottom to allow leachate of water from plants drain into the mesocosms during precipitation. The holes were covered with gauze (diameter: 7 mm; 200 µm) to prevent that larger particles from the soil could fall into the mesocosms. On top of each mesocosm, three flowerpots were installed. Six plants of *I. glandulifera* were planted in each flowerpot representing the *Impatiens* treatment (total: 18 plants). Flowerpots without plants were installed in the control treatments. *Impatiens* treatment and control were randomly assigned to the mesocosms in the experimental area. In order to avoid differences due to shading, the controls were provided with camouflage nets (“Universalnetz”). The soil for the *Impatiens* treatment and controls was taken from the ground of a freshwater basin from the ecological botanical garden in Bayreuth (49°92´45”N, 11°58´56”E). The soil from the same batch was randomly distributed either to the control or the *Impatiens* treatment. *I. glandulifera* (size: 30 – 50 cm) were collected from one riparian zone of the Red Main river in Bayreuth, Germany (49°95´64”N, 11°56´29”E) in May 2016. One week before the experiment, the 24 mesocosms were filled with 750 L tap water. Since tap water contains only low levels of trace elements and is therefore not an optimal medium to culture daphnids, we enriched the medium of the mesocosms with sea‐salt, CaCl_2_, SeO_2_, and phosphate‐buffer, a standard procedure to prepare a semi‐artificial medium for *Daphnia* culture (Rabus & Laforsch, 2011; Table S7). Furthermore, 3 L of pooled green algae *A. obliquus* (0.11 Pigment (Chl‐a)/m^3^) were added to each tank. At the first of June 2016, we added 195 non‐mature pooled *D. magna* individuals, 15 individuals of each clone, to each the mesocosms.

The mesocosms were sampled (*Daphnia* +Chlorophyll‐a) weekly after homogenization using a plastic stick (stirring three times within the form of lying eight and once centrically) at Wednesday mornings. The chemical and physical parameters (temperature, pH, oxygen concentration, and conductivity) were measured with a multiparameter meter probe (HI 9829, Hanna Instruments Deutschland GmbH, Voehringen, Germany; Figure S1). The rainfall was recorded in a close by weather station in the botanical garden of the University of Bayreuth. To determine the phytoplankton concentration, we took a volume of 500 mL water from each mesocosm after homogenization and measured the chlorophyll‐a concentration following the method by Parsons and Strickland (Strickland & Parsons, 1972). To determine the population density of daphnids, we performed a vertical net haul after homogenization at the center of the mesocosms using a self‐made plankton net (height: 8.2 cm, width: 4.6 cm covered by gauze (130 µm); total sample volume: 8.4 l water). Subsequently, samples were conserved using 70%‐EtOH. All *D. magna* individuals were counted using a dissecting microscope (Magnification: 1.6 x; Leica M50, Leica Microsystems GmbH, Wetzlar, Germany; equipped with an Olympus DP26 camera, Olympus Deutschland GmbH, Hamburg, Germany; CellSens Dimension v. 1.11, Olympus Deutschland GmbH, Hamburg, Germany).

### 2.5 Acute toxicity test with *D. magna*

All experiments were carried out with animals from one *D. magna* (K34J) clone. Six different 2‐MNQ (1, 2, 3, 6 and 12 mg/L) concentrations, the corresponding solvent controls, and a control treatment were tested. Five neonate *Daphnia* (<24 h old), were randomly placed into each 50 mL beaker. The animals were not fed during the test. Each test solution was replicated four times with temperature and photoperiod controlled as described above. Mortality was recorded after 24 and 48 h, when the experiment was terminated.

### 2.6 Chronic toxicity test with *D. magna*

The test was carried out with animals from one *D. magna* (K34J) clone. Chronic toxicity tests of non‐lethal 2‐MNQ concentrations to *D. magna* followed the procedures recommended by the OECD guideline Nr. 211(OECD, 2011). Three different 2‐MNQ (0.075, 0.75, and 1.5 mg/L) concentrations, the corresponding solvent controls (DMSO: 100 µl, 50 µl, 0.5 µl), and a control treatment was used. Neonates (age <24 h) from the fourth generation of a pool of females from the *D. magna (K34J)* clone were randomly placed into the experimental vials. Twenty replicates with one neonate per vial (160 mL, WECK GmbH, Wehr‐Oeflingen, Germany) were used for each of the seven treatments. Animals were transferred daily to fresh media (test solution +1 mg *A. obliquus*), to ensure that the animals were not food limited and to keep the 2‐MNQ concentration on a stable level. The effects of 2‐MNQ on age at maturity, body size at maturity (first time freshly deposited eggs visible), and on the total number of offspring were determined. Brood size was determined whenever the offspring was released from the brood pouch. Morphological parameters (body size, body width, and tail‐spine length) were measured as described by Trotter et al. (2019), using a stereomicroscope equipped with a digital image‐analysis system (Leica M50, Leica Microsystems GmbH, Wetzlar, Germany; equipped with an Olympus DP26 camera, Olympus Deutschland GmbH, Hamburg, Germany; CellSens Dimension v. 1.11, Olympus Deutschland GmbH, Hamburg, Germany). The test was terminated after day 21, following the OECD guideline Nr. 211 (OECD, 2011).

### 2.7 Growth test of *A. obliquus*

At the start of the test, a volume of 400 µl algae solution of *A. obliquus* with a concentration of 2.3*10^5 cells/ml (Zehnder Z/4 medium) was transferred into each well of a 96‐well plate (Eppendorf AG, Hamburg, Germany). Three different 2‐MNQ (1.5, 3, and 6 mg/L) concentrations were added at the beginning of the experiment. Each 2‐MNQ treatment and a control treatment without the addition of 2‐MNQ was replicated seven times. The plate was shaken on a horizontal shaker (IKA‐ViBRAX‐VXR (IKA^®^‐Werke GmbH & CO. KG, Germany); Speed = 180 rpm) and the light source (Osram Lumilux CoolWhite, 58 W/840; Osram GmbH, München) was placed above (h = 40 cm) the well plates, with a 21:3 day/night cycle at 20°C. The absorbance at 800 nm, as marker for the cell number of *A. obliquus*, was measured using a plate reader (Synergy HT, BioTek Instruments, Inc., Winooski, USA), at the beginning of the experiment, after 24, 48, and 96 h.

### 2.8 Measurement of oxidative stress in *A. obliquus*

In order to characterize a potential stress response in *A. obliquus* when exposed to 2‐MNQ, we tested for an increase of reactive oxygen species (ROS) formation. Since population growth of *A. obliquus* was already decreased at a concentration of 1.5 mg/L 2‐MNQ (see below in results; Figure 4), we used concentrations of 0.075, 0.75, and 1.5 mg/L 2‐MNQ, the same 2‐MNQ concentrations as used in the chronic exposure test with *D. magna*. The general content of ROS was measured using the fluorescence dye 2’,7’‐dichlorofluorescin diacetate (DCFDA) (CAS Number: 4091‐99‐0, Merck KgaA, Darmstadt, Germany), which was prepared and diluted with algae media (Z/4) to a final concentration of 80 µmol. For the study we used 50 µL algal solution with a concentration of *A. obliquus* of 2.3 × 10^5^ cells/ml which were cultivated on the same medium as previously described, with a 21:3 day/night cycle at 20°C.

The study followed the method from Knauert and Knauer (2008): In brief, a 96‐well plate (Eppendorf AG, Hamburg, Germany) was prepared with three control treatments (algae, algae‐media, dye and algae medium; 2 replicates each) and five different dye treatments (Control (only Z/4 media), 100 µl DMSO, 0.075 mg/L 2‐MNQ, 0.75 mg/L 2‐MNQ, 1.5 mg/L 2‐MNQ; 18 replicates each). At first 50 µL of each dye treatment (control, DMSO, 2‐MNQ), followed by 100 µL dye medium and finally 50 µL algae solution were added to a final volume of 200 µL. The plate was then placed in the dark at room temperature (22°C) for 30 min to ensure that the dye can enter the algae cells. Afterward, the light was started. The fluorescence was measured (Synergy HT, BioTek Instruments, Inc.; λ_ex_ = 485 nm, λ_em_ = 529 nm) every 30 min for 4.5 h. In the meantime, the algae were gently mixed on a horizontal shaker (IKA‐ViBRAX‐VXR, IKA^®^‐Werke GmbH & CO.); Speed = 300 rpm) at 25°C under constant light conditions (867.5 Lux).

### 2.9 Statistical analysis

#### 2.9.1 Mesocosm study with *D. magna* and *A. obliquus*

We calculated the mean population density and chlorophyll‐a content from all replicas (*n* = 12) per treatment and used a one‐way repeated measures ANOVA to compare differences between the weeks and the *I. glandulifera* treatment and control (RM – ANOVA, SPSS, version 21, SPSS Inc., Chicago, IL, U.S.A.). When the sphericity was not met a Greenhouse‐Geisser correction of sphericity was used.

#### 2.9.2 Acute toxicity tests with 2‐MNQ and *D. magna* and *A. obliquus*

We calculated the dose‐response curves of all endpoints (*D. magna*: 48 h; *A. obliquus*: 96 h) with R (v. 2021.09.1 Build 372) and the drc package (version 3.0‐1) (Ritz et al., 2015). To determine the best curve fit and the EC50 value, different models (Log‐logistic (three, four, and five parameter) and Weibull (three, four, and five parameter)) were compared, and the best fit was chosen by the lowest lack of fit value.

#### 2.9.3 Chronic toxicity test with 2‐MNQ and *D. magna*

To determine differences between the treatments, we used the software package SPSS v21 (SPSS Inc). The data were tested for normal distribution (Shapiro Wilk) and checked for homogeneity of variances (Levene‐test). Since one of these assumptions was not met (Tables S3 and S4), a non‐parametric Kruskal‐Wallis test with a Dunn‐Bonferroni Post‐hoc test was conducted. Furthermore, we calculated the dose‐response curves of all endpoints with R (v. 2021.09.1 Build 372) and the drc package (version 3.0–1). To determine the best curve fit and the EC50 value, different models (Log‐logistic (three, four, and five parameter) and Weibull (three, four, and five parameter)) were compared, and the best fit was chosen by the lowest Lack of fit value. Possible hormetic responses were determined by comparing the Brain‐Cousens model with the used model for the EC50 calculation.

#### 2.9.4 Measurement of ROS in *A. obliquus*

For the ROS test in *A. obliquus*, we calculated the mean from all replicates (*n* = 18) for each treatment and used a one‐way repeated measures ANOVA to compare between time and different treatments (control, solvent‐control, 2‐MNQ‐Treatments: 0.075, 0.75, 1.5 mg/L) (RM – ANOVA, SPSS, v21, IBM, Chicago, IL, U.S.A.).

## 3 RESULTS

### 3.1 Mesocosm study

**FIGURE 2:**
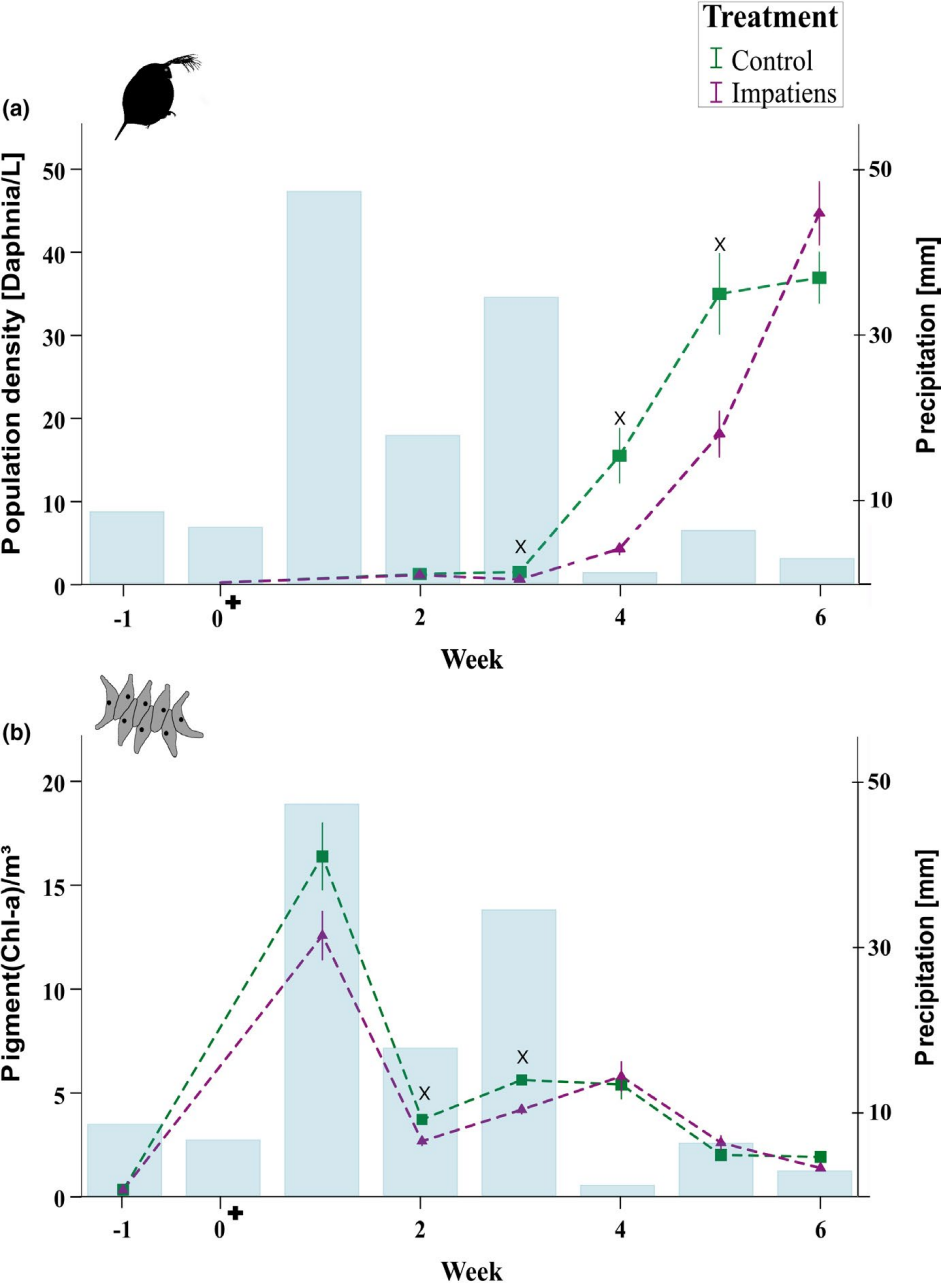
*Daphnia magna* population density (measured in *Daphnia* per liter) as proxy for *Daphnia* population growth, and chlorophyll‐a content (measured in pigment chlorophyll‐a/l) as proxy for algae population density within the mesocosm study. Blue bars show the weekly precipitation. The plus indicates the start of the experiment, when *I. glandulifera* was placed above each mesocosm. Green squares show the mean population density of the control mesocosms while the purple triangles show the mean population density of the mesocosms treated with *I. glandulifera* (*n* = 12). The x indicates a significant difference between treatment and control. (a) *D. magna* population density (*Daphnia*/l): Population density of *D. magna* was significantly lower in the mesocosms exposed to leachate of *I. glandulifera* compared to the controls, from the third to the fifth's week, indicating that reduced population growth in *Daphnia* is a time‐delayed response to allelochemicals introduced into the mesocosms after precipitation. All points show means ±standard error (SEM). (b) *A. obliquus* was added one week before the experiment started (indicated on x‐axis: −1), to ensure sufficient food supply for *D. magna*. Compared to the time‐delayed response observed in *D. magna*, leachate from *I. glandulifera* inhibits the growth of the algae *A. obliquus* immediately in periods of heavy rainfall. While in the first experimental week *A. obliquus* populations exposed to *I. glandulifera* showed only a trend toward a reduction compared to the controls, it was significantly reduced in the second and third week. All points show means ±standard error (SEM)

Overall, the population density of *D. magna* increased significantly per week in both the *I. glandulifera* treated and control mesocosms (repeated measures ANOVA, *F*_(2.4, 51.65)_ = 7.51; *p* = .001, corrected using Greenhouse–Geisser estimates of sphericity). However, the population growth of *D. magna* was significantly reduced by the presence of *I. glandulifera* in comparison to controls after a period of strong precipitation (Figure 2a; repeated measures ANOVA, *F*_(1,22)_ = 4.92; *p* = .035, corrected using Greenhouse–Geisser estimates of sphericity). In particular, population density of *D. magna* was significantly lower in the mesocosms exposed to leachate of *I. glandulifera* compared to the controls, from the third to the fifth week (Figure 2a; Week 3: *df* = 22, *p* = .002; Week 4: *df* = 22, *p* = .007; Week 5: *df* = 22, *p* = .008). The population density of *A. obliquus* changed significantly per week in both the *I. glandulifera* treated and control mesocosms (repeated measures ANOVA, *F*_(1.86, 35.35)_ = 111.03; *p* ≤ .001, corrected using Greenhouse–Geisser estimates of sphericity). The population growth of *A. obliquus* was reduced immediately in periods of heavy rainfall in the mesocosms treated with *I. glandulifera* compared the non‐treated mesocosms (Figure 2b, week 1–3). Within the first week of the experiment, we observed a trend towards a reduction in the chlorophyll‐a content in mesocosms treated with *I. glandulifera* compared to the control (unpaired Student´s *t*‐test, *df* = 20, *p* = .069). In the second (unpaired Student´s *t*‐test, *df* = 22, *p* = 0.003) and third week (unpaired Student´s *t*‐test, *df* = 21, *p* = .035) of the experiment the growth of *A. obliquus* was significantly reduced. Over the entire time period of the experiment, *A. obliquus* shows only a trend toward a reduced growth in mesocosms treated with *I. glandulifera* leachate in comparison to controls, since in periods of no or low rainfall no difference between both treatments could be observed (Figure 2b; repeated measures ANOVA, test for between‐subject effects, *F*_(1;88.19)_ = 3.14; *p* = .059).

### 3.2 Laboratory studies on effects of 2‐MNQ on *D. magna*

**FIGURE 3:**
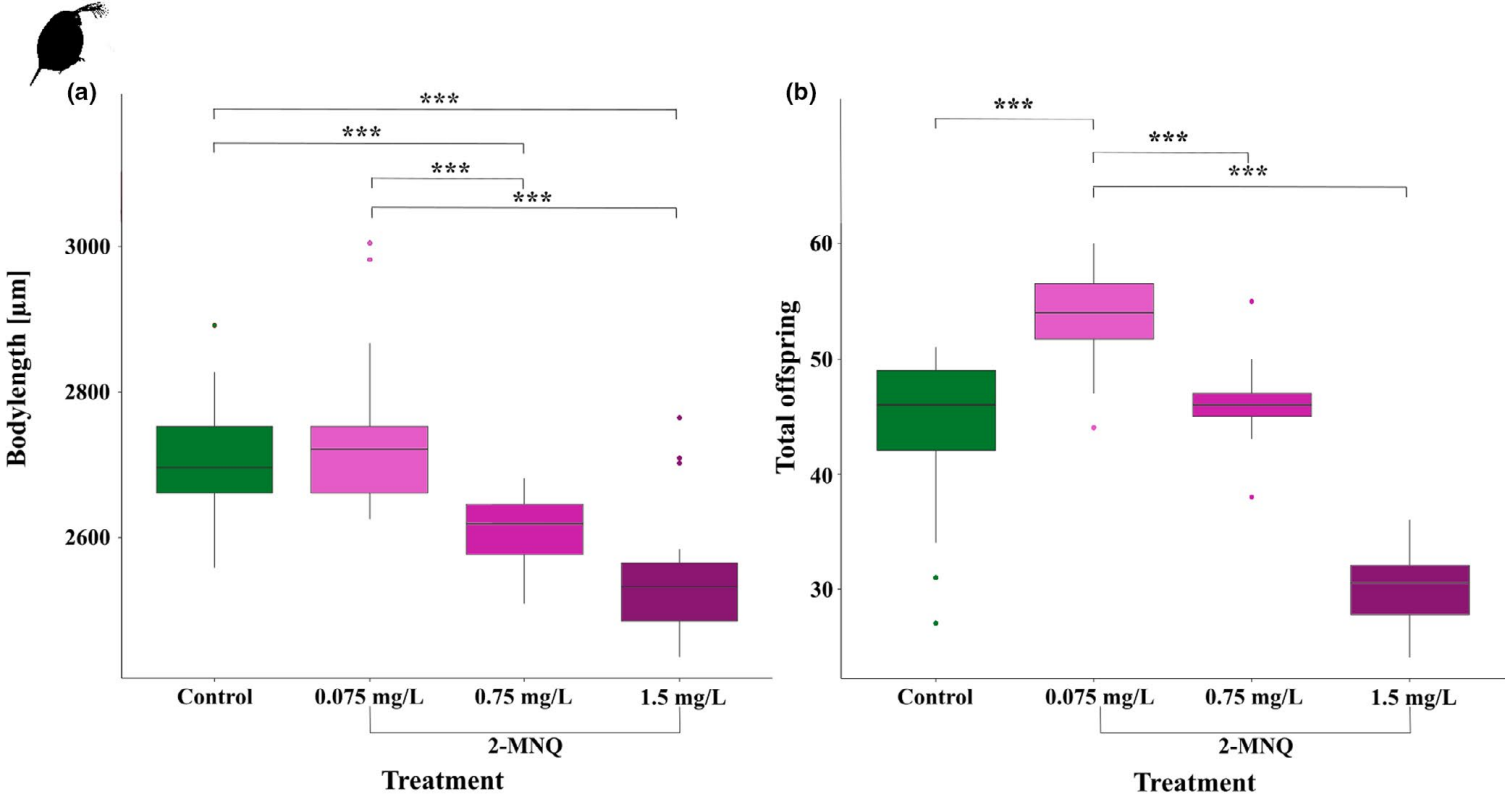
Effects of 2‐MNQ on body length and reproduction in *Daphnia magna* during the 21‐day chronic toxicity test. Control: green; 2‐MNQ: 0.075 mg/L, light purple; 0.75 mg/L, mid‐purple; 1.5 mg/L, dark purple; *n* = 20. (a) Body length at maturity: When exposed to 0.75 mg/L 2‐MNQ and 1.5 mg/L 2‐MNQ the animals were significantly smaller compared to the control and the 0.075 mg/L 2‐MNQ treatment at maturity. (b) Total number of offspring produced during the chronic toxicity test: When exposed to 2‐MNQ animals treated with 1.5 mg/L had significantly fewer offspring, while when treated with 0.075 mg/L the animals had significantly more offspring

To determine the lethal threshold of 2‐MNQ on *D. magna* we conducted an acute toxicity test. We determined an EC50 of 2.84 mg/L (95%‐ Confidence interval: 2.809 and 2.869 mg/L) (dose‐response curve, Figure S2a). Based on these results, we selected three sub‐lethal 2‐MNQ concentrations (0.075, 0.75, and 1.5 mg/L) for a 21‐day subsequent chronic toxicity test. During the test, we exchanged the medium and algae daily to prevent indirect effects from reduced food availability. We could show that a long‐term exposure with 2‐MNQ had significant negative effects on body size and reproduction of *D. magna* (Figure 3a; Body size: Kruskal‐Wallis test with a Bonferroni corrected Dunn's post hoc test: *H* = 42.4, *p* ≤ .001 and EC50 = 0.649 (95%‐ Confidence interval: 0.299 and 0.999 mg/L, dose‐response curve Figure S2b); Figure 3b Reproduction: Kruskal‐Wallis test with a Bonferroni corrected Dunn's post hoc test: *H* = 56.16, *p* ≤ .001 and EC50 = 1.690 mg/L (95%‐ Confidence interval: 1.554 mg/L and 1.827 mg/L, Dose‐response curve Figure S2c)). Compared to individuals exposed to the control medium and to 0.075 mg/L 2‐MNQ, animals exposed to higher concentrations of 2‐MNQ (0.75 mg/L and 1.5 mg/L) had a significantly reduced body size at maturity (Figure 3a; *P*_(C–0.75)_ = 0.001; *P*_(C–1.5)_ < 0.001; *P*_(0.075–0.75)_ = 0.001; *P*_(0.075‐1.5)_ < 0.001). Furthermore, concentrations higher than 0.75 mg/L 2‐MNQ led to significantly fewer offspring in *D. magna* compared to all other treatments (Figure 3b; *P*_(C‐0.075)_ = 0.002; *P*_(C‐1.5)_ = 0.001; *P*_(0.075–0.75)_ = 0.006; *P*_(0.075–1.5)_ < 0.001; *P*_(0.75–1.5)_ < 0.001). In contrast, animals treated with 0.075 mg/L 2‐MNQ showed the highest number of offspring compared to all other treatments (Figure 3b; *P*_(C‐0.75)_ = 0.001; *P*_(C‐1.5)_ < 0.001; *P*_(0.075–0.75)_ = 0.001; *P*_(0.075–1.5)_ < 0.001). This effect was a hormetic response, because the Brain‐Cousens model from (van Ewijk & Hoekstra, 1994) differed significantly from the curve fit (Figure S2c; One‐way ANOVA: *F* = 39.034; *p* < .001).

### 3.3 Laboratory studies on effects of 2‐MNQ on *A. obliquus*

**FIGURE 4:**
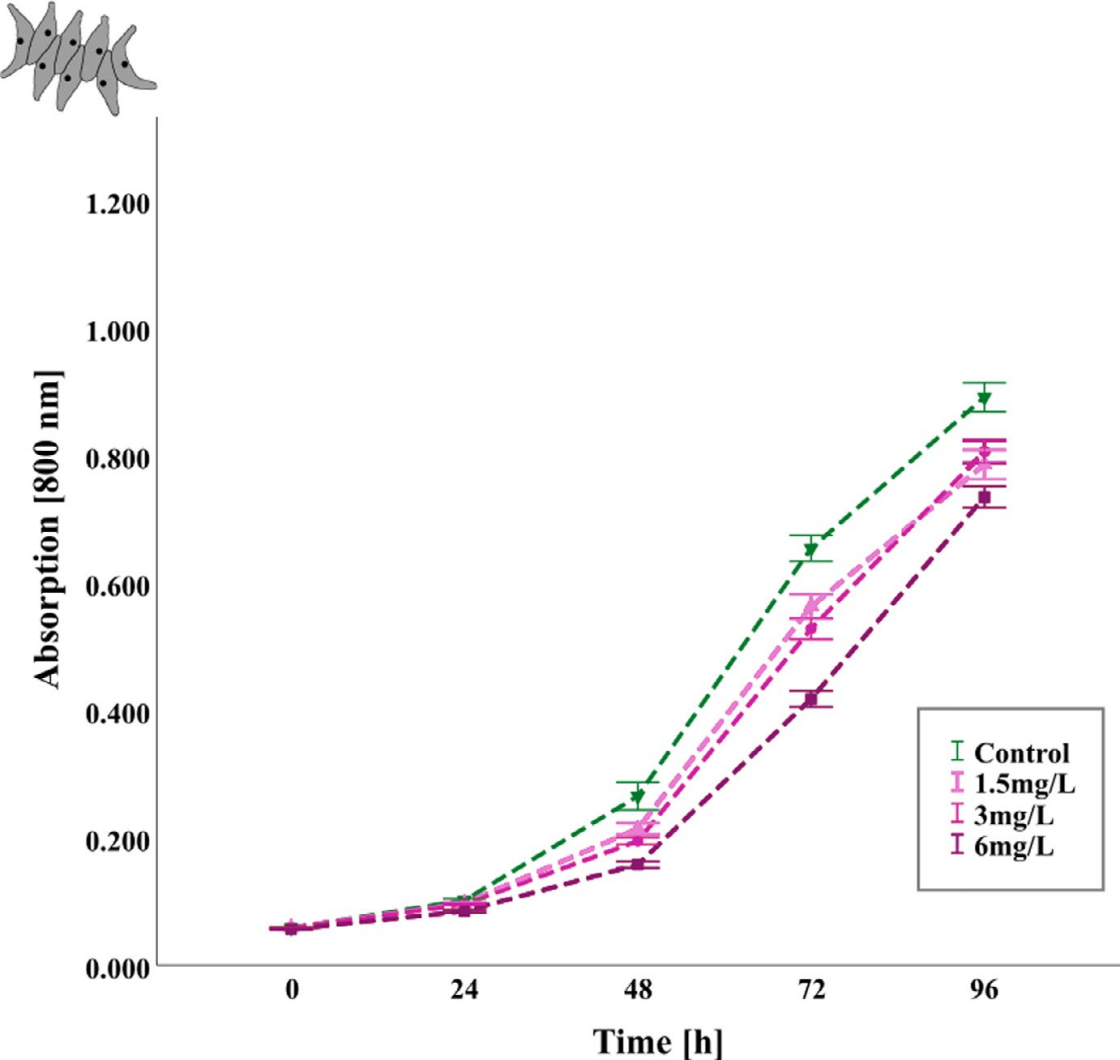
Effects of 2‐MNQ on the population growth of *Acutodesmus obliquus* (measured as absorption of light at 800 nm wavelength) over 96 h. Control (green), 1.5 mg/L 2‐MNQ (light‐purple), 3 mg/L 2‐MNQ (mid‐purple), 6 mg/L 2‐MNQ (dark‐purple); *n* = 18. When exposed to 2‐MNQ the growth rate of *A. obliquus* was significant. Each data point represents the mean ±standard error (SEM)

To test for direct toxic effects of *I. glandulifera* on the green alga *A. obliquus*, which may also account for reduced food availability for *D. magna*, we used three concentrations of 2‐MNQ (1.5, 3, and 6 mg/L). Our results show that a pulsed concentration of 2‐MNQ, as given after precipitation in the mesocosm study, significantly reduced population growth of *A. obliquus* over 96 h in all three tested concentrations compared to the control treatment (Figure 4; repeated measures ANOVA, *F*_(3.00, 24.00)_ = 21.47; *p* < .001; *P*_(C‐1.5)_ = 0.047; *P*_(C‐3)_ = 0.009; *P*_(C‐6)_ < 0.001). Overall, algal growth (measured as absorption of light at 800 nm wavelength) changed significantly over time in all treatments and the controls (repeated measures ANOVA, *F*_(4.55, 36.36)_ = 17.74; *p* < .001, corrected using Greenhouse–Geisser estimates of sphericity). Furthermore, we determined an EC50 of 10.687 mg/L (Figure S2d; 95%‐ Confidence interval: 6.467 and 14.9074 mg/L).

**FIGURE 5:**
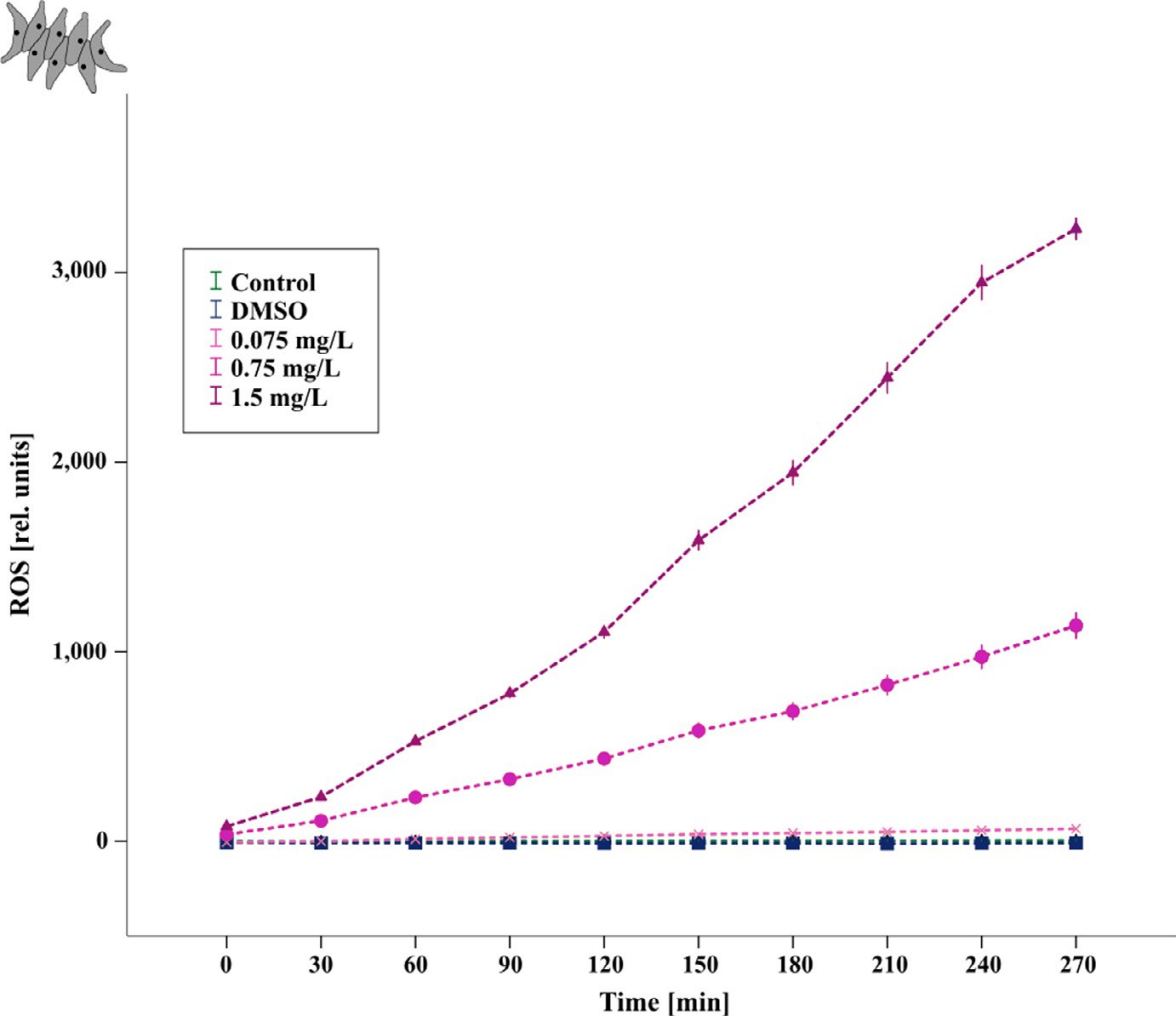
Total ROS formation (expressed as fluorescence units) in the green alga *Acutodesmus obliquus* upon exposure to 2‐MNQ. Control (green), 0.075 mg/L 2‐MNQ (light‐purple), 0.75 mg/L 2‐MNQ (mid‐purple), 1.5 mg/L 2‐MNQ (dark‐purple); *n* = 18. When treated with 2‐MNQ all algae cells showed a significant increase in the production of ROS. Each data point represents the mean ±standard error (SEM)

Concerning the ROS production we found a significant time‐treatment interaction (repeated measures ANOVA, *F*_(1.4, 120.42)_ = 23932.95; *p* < .001, corrected using Greenhouse–Geisser estimates of sphericity). Further, ROS production in the algae increased significantly with increasing concentrations of 2‐MNQ (Figure 5; repeated measures ANOVA, *F*_(4,85)_ = 11803.751; *p* < .001). All tested 2‐MNQ concentrations showed significantly higher ROS production than the control groups (*P*_(C–0.075)_ = 0.009; *P*_(C–0.75)_ < 0.001; *P*_(C–1.5)_ < 0.001; *P*_(DMSO–0.075)_ < 0.001; *P*_(DMSO–0.75)_ < 0.001; *P*_(DMSO‐1.5)_ < 0.001).

## 4 DISCUSSION

Our results indicate that leachate from an invasive terrestrial plant has negative impacts on organisms across ecosystem boundaries. We show that the leachate and the pure allelochemical 2‐MNQ of the invasive terrestrial plant *I. glandulifera* have direct negative effects on two model organisms of different trophic levels in an aquatic ecosystem, the alga *A. obliquus*, and the water flea *D. magna*. Concentrations of 2‐MNQ far lower than the highest measured concentration of leached 2‐MNQ during precipitation (12 mg/L) (Ruckli et al., 2014) are already leading to significant negative responses in both species.

This indicates that even if 2‐MNQ is diluted when it leaches into aquatic ecosystems, it may still have consequences for this system, especially when monospecific stands of *I. glandulifera* are present. This is supported by the results of our mesocosm study, where we could show that after precipitation the population density of *A. obliquus* and *D. magna* were reduced. A recent study showing that during rainfall the concentration of secondary plant metabolites was about ten times higher in surface water of streams further support our findings (Griffiths et al., 2021; Günthardt et al., 2020; Kisielius et al., 2020). Unfortunately, we could not reliably measure the concentration of 2‐MNQ in the mesocosms. Although we do not know the exact concentration of the allelochemicals responsible for the observed effects, reduced population growth in both species only occurred in mesocosms exposed to *I. glandulifera* after precipitation.

This fact strongly indicates that leachates from this invasive plant also affect aquatic organisms. Further, in natural ecosystems, it is likely that the concentration of 2‐MNQ may additionally increase due to the introduction of plant material and accumulation of 2‐MNQ in sediments (Kessler, 1989). A similar accumulation in the aquatic environment has been shown for other phenolic compounds (Armstrong & Boalch, 1961; Chen & Gardner, 2004; Lambdon et al., 2008; McLachlan & Craigie, 1964). In addition, naphthoquinones have high persistence time in water, e.g., juglone was shown to have a half‐life in non‐sterile pond water of 87 ± 7.4 h (Wright, Dawson, et al., 2007). Therefore, it is not expected that the concentration of 2‐MNQ will rapidly decline after its introduction in freshwater ecosystems.

Naphthoquinones, like 2‐MNQ, induce the formation of reactive oxygen species and thus cause oxidative stress and may affect redox signaling in exposed tissues (Klotz et al., 2014). Thus, the observed negative effects of 2‐MNQ to both organisms in our study may be due to oxidative stress caused by the substance.

In *D. magna*, 2‐MNQ affects important life history and morphological traits, such as number of offspring and body size. Reduced body size is not only correlated with fewer offspring and therefore reduced population growth in *Daphnia* (Lürling et al., 2006), but also renders them more vulnerable to predatory invertebrates (Riessen et al., 2012), since smaller Daphnids would be an easier prey for size‐dependent predators like the phantom midge, *Chaoborus* sp. (Pastorok, 1981; Young & Riessen, 2005). Further, exposure to allelochemicals released by *I. glandulifera* may elicit maternal effects, meaning that the phenotype of the mother affects the phenotype of her offspring (Agrawal et al., 1999; Frost et al., 2010). This may lead to a reduced body size, including the above‐mentioned consequences, for several generations. As a result, even a pulsed leachate from *I. glandulifera* may have long‐term implications on the fitness of this keystone species of ponds and lakes, as indicated by the results of the mesocosm study. Here, the population growth rate of *Daphnia* exposed to leachate from *I. glandulifera* was reduced for weeks, even after a short period of precipitation. This may have consequences for the entire food web since the primary consumer *Daphnia* acts as an important link between primary producers and higher trophic levels (Sommer & Stibor, 2002). Especially young‐of‐the‐year fishes are affected by the decline of zooplankton, because they depend on this food resource in early summer (Lampert, 1993).

The increased fertility in *D. magna* observed in the lowest 2‐MNQ (0.075 mg/L) treatment in the laboratory study indicates a hormesis effect, i.e., an often reported stimulating effect of toxins at a very low dose (Flaherty & Dodson, 2005). This response to low concentrations of 2‐MNQ may at the same time have appreciable metabolic costs, as discussed in other studies (Chapman, 2002). Since population growth rate in the mesocosm study was significantly reduced when exposed to leachate from *I. glandulifera* this effect observed in the laboratory does not seem to be relevant for natural populations.

In the primary producer *A. obliquus*, a pulsed concentration of 2‐MNQ was sufficient to significantly reduce population growth in an acute 96 h toxicity test. This is in accordance with the pattern observed for chlorophyll‐a concentration in the mesocosm study. Shortly after precipitation, the population growth in *A. obliquus* was significantly reduced when exposed to leachate from *I. glandulifera*. A reduction in algae population growth can also occur due to a lack of nutrients since we did not fertilize the mesocosms. However, the chlorophyll‐a content in the control treatments without *I. glandulifera* was still higher. This strongly indicates that the leachate from *I. glandulifera* reduces population growth in *A. obliquus*.

As a consequence, inhibition of algae growth reduces food availability for *D. magna*, which may amplify the direct negative effects of *I. glandulifera* on this species. The reduced food concentration therefore results in a further reduction of the population size of *Daphnia*, as observed in the mesocosms after a short lag‐phase subsequent to precipitation. In addition, former studies showed that the fitness of daphnids is even reduced when only stressed algae are available as food source (De Lange & Van Reeuwijk, 2003). Such animals have an increased age at first reproduction and a reduced clutch size. Our laboratory study shows that all tested concentrations of 2‐MNQ increase ROS production in algae. This increased ROS level can change the biochemical composition in algae cells, which reduces food quality for *Daphnia* (Pinto et al., 2003). It is therefore likely that direct effects of *I. glandulifera* on both food quantity and quality of *A. obliquus* could have indirectly affected *D. magna* population growth in the mesocosm study and may reduce the growth of *D. magna* populations in natural ecosystems as well (Sikora et al., 2014). In addition, the EC50 values indicate that *D. magna* is more sensitive to 2‐MNQ compared to *A. obliquus* in the acute tests. However, this might have to do with the reactivity of 2‐MNQ, because it was shown that naphthoquinones are photosensitive and the algae were treated 96h in a static test (Wright, Dawson, et al., 2007).

Hence, both direct and indirect effects of leachate from *I. glandulifera* may manifest gradually in organisms and populations, ultimately resulting in ecosystem changes. The observed reduction in population growth rates after pulses of 2‐MNQ after precipitation may result in cascading effects from lower trophic levels such as cladocerans and rotifers (Hanazato, 2001) to higher trophic levels such as fish (Hanazato, 2001; Havens, 1998). This may affect the entire food web in aquatic ecosystems which are surrounded by monospecific stands of *I. glandulifera* in the riparian zone. These cascading effects might extend to trophic levels, relevant for fishing industry and human consumption (Cardinale et al., 2012).

This demonstrates that for *I. glandulifera*, although already being evaluated as an invasive plant by governmental institutions, a comprehensive understanding of the far‐reaching impacts across ecosystem‐boundaries has still not been fully achieved. Recent studies showed that natural waterbodies may contain a cocktail of other phytotoxins, like coumarins, formononetin and alkaloids in concentrations of up to 90 µg/L (Günthardt et al., 2021; Nanusha et al., 2020, 2021; Nanusha, Krauss, Schönsee, et al., 2020), which may act synergistically, and further amplify the effects of low doses of 2‐MNQ on aquatic ecosystems. Our study paves the way to better assess the hazard of invasive terrestrial plant species on aquatic ecosystems under both: natural‐like and laboratory conditions. We show that the across ecosystem effects are more potent than expected and future risk assessment studies on terrestrial invasive plants need to address this aspect.

## CONFLICT OF INTEREST

The authors declare no conflict of interests.

## AUTHOR CONTRIBUTION

**Jens Georg Peter Diller:** Conceptualization (equal); Funding acquisition (equal); Investigation (equal); Methodology (equal); Visualization (equal); Writing – original draft (equal). **Sophia Drescher:** Conceptualization (equal); Investigation (equal); Methodology (equal). **Mario Hofmann:** Conceptualization (equal); Investigation (equal); Methodology (equal). **Max Rabus:** Conceptualization (equal); Investigation (equal); Methodology (equal). **Heike Feldhaar:** Conceptualization (equal); Methodology (equal); Writing – original draft (equal); Writing – review & editing (equal). **Christian Laforsch:** Conceptualization (equal); Funding acquisition (equal); Methodology (equal); Writing – original draft (equal); Writing – review & editing (equal).

## Supporting information

Supplementary Material

## Data Availability

Raw data can be accessed via Dryad Digital Repository (https://datadryad.org/stash/share/5mCOg3pzVRNRHDFFcD8DVusliHML69afSWY8eDMD1Rk).
