# Supplementary material for: The Beauty is a beast: Does leachate from the invasive terrestrial plant Impatiens glandulifera affect aquatic food webs?
Source: Ecol Evol. 2022 Apr 6;12(4):e8781. doi: 10.1002/ece3.8781 (PMC8986513; doi:10.1002/ece3.8781)
Supplement: Supplementary file 1 — Supplementary Material [file ECE3-12-e8781-s001.docx]

**Supplementary Material: The Beauty is a beast: Does leachate from the invasive terrestrial plant *Impatiens glandulifera* affects aquatic food webs?**

Authors: Jens G. P. Diller^1,2^, Sophia Drescher^1^, Mario Hofmann^1^, Max Rabus^1,2^, Heike Feldhaar^1,2^ and Christian Laforsch^1,2^

^1^Animal Ecology I, Universitaetsstraße 30, 95447 Bayreuth

^2^Bayreuth Center for Ecology and Environmental Research (BayCEER), Universitaetsstraße 30, 95447 Bayreuth


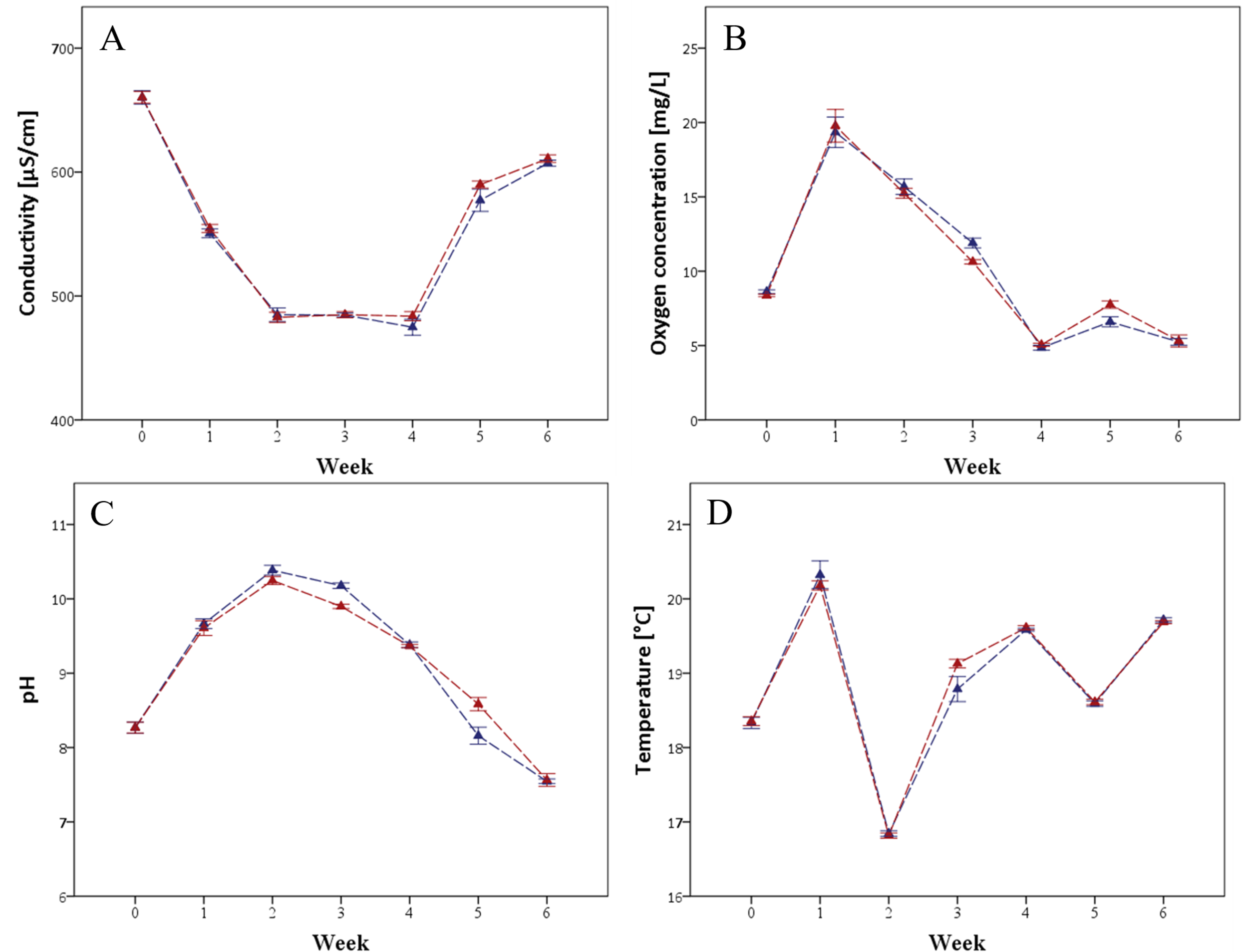


**Figure S1: Physical/chemical parameters during the mesocosm study**. The red dotted line shows the trend for I. glandulifera and the blue dotted line for the control for conductivity (A), oxygen concentration (B), pH (C) and temperature (D).

**Table S1: Chemical Structure and Physical/Chemical Parameters of 2-MNQ**

| 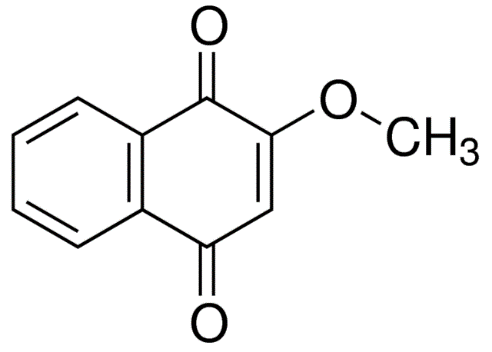 | MW = 188.19 g/mol |
| --- | --- |
|  | LogKow = 1.13 |
|  | Melting Point = 183 °C |
|  |  |


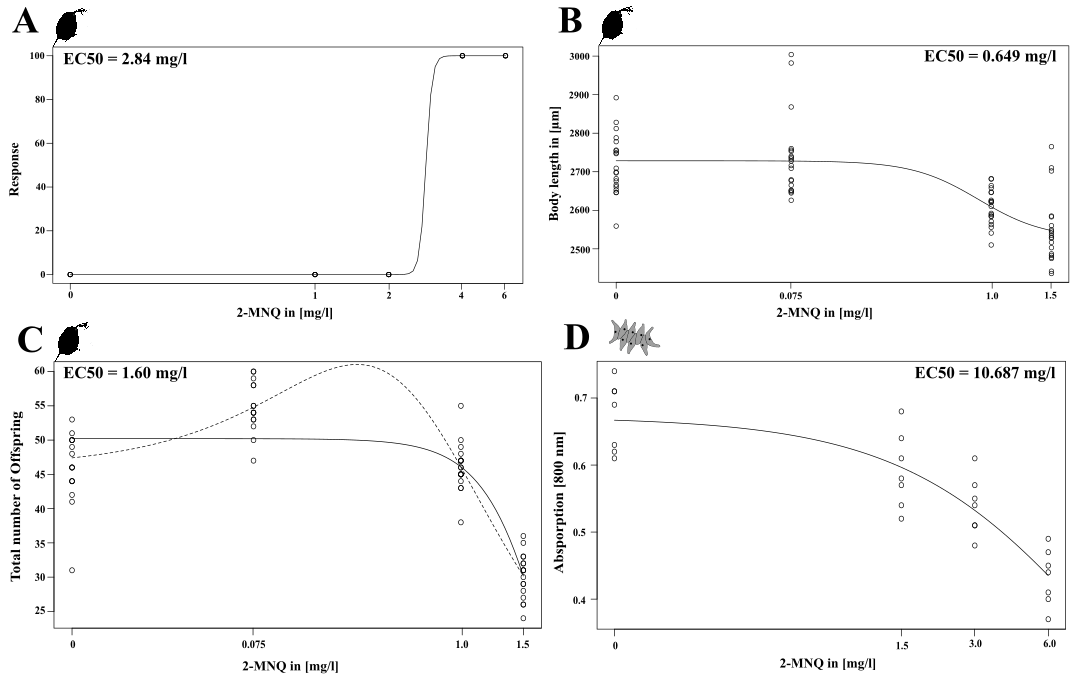


**Figure S2: Dose-response curves of Daphnia magna and Acutodesmus obliquus to 2-MNQ.** A) Mortality of D. magna after 48 h to different concentrations of 2-MNQ (1, 2, 4 and 6 mg/L). B) Body length of D. magna at primiparity to different 2-MNQ concentrations (0.075, 0.75, and 1.5 mg/L). C) Total offspring with hormesis curve (dashed line) of D. magna to different 2-MNQ concentration (0.075, 0.75, and 1.5 mg/L). D) Response of A. obliquus to different 2-MNQ concentrations (1.5, 3, 6 mg/L).

**Table S2: Post hoc tests for measured body size of D. magna during the chronic toxicity test.** C (control); 0.075 mg/l 2-MNQ concentration; 0.75 mg/l 2-MNQ concentration; 1.5 mg/l 2-MNQ concentration

| Sample 1-Sample 2 | Test Statistic | Std. Error | Std. Test Statistic | Sig. | Adj. Sig. |
| --- | --- | --- | --- | --- | --- |
| 1.5 2MNQ-0.75 2MNQ | 11.450 | 7.348 | 1.558 | .119 | .715 |
| 1.5 2MNQ-Control | 38.375 | 7.348 | 5.223 | **.000** | .000 |
| 1.5 2MNQ-0.075 2MNQ | 38.775 | 7.348 | 5.277 | **.000** | .000 |
| 0.75 2MNQ-Control | 26.925 | 7.348 | 3.664 | **.000** | .001 |
| 0.75 2MNQ-0.075 2MNQ | 27.325 | 7.348 | 3.719 | **.000** | .001 |
| Control-0.075 2MNQ | -.400 | 7.348 | -.054 | .957 | 1.000 |
| The significance level is .05. | | | | | |

**Table S3: Post Hoc tests for total offspring of D. magna during the chronic toxicity test:** C (control); 0.075 mg/l 2-MNQ concentration; 0.75 mg/l 2-MNQ concentration; 1.5 mg/l 2-MNQ concentration

| Sample 1-Sample 2 | Test Statistic | Std. Error | Std. Test Statistic | Sig. | Adj. Sig. |
| --- | --- | --- | --- | --- | --- |
| 1.5 2MNQ-Control | 27.256 | 7.355 | 3.706 | **.000** | .001 |
| 1.5 2MNQ-0.75 2MNQ | 29.925 | 7.159 | 4.180 | **.000** | .000 |
| 1.5 2MNQ-0.075 2MNQ | 53.575 | 7.159 | 7.484 | **.000** | .000 |
| Control-0.75 2MNQ | -2.669 | 7.355 | -.363 | .717 | 1.000 |
| Control-0.075 2MNQ | -26.319 | 7.355 | -3.578 | **.000** | .002 |
| 0.75 2MNQ-0.075 2MNQ | 23.650 | 7.159 | 3.304 | **.001** | .006 |
| The significance level is .05. | | | | | |

**Table S4: Post hoc tests for growth rate of A. obliquus with absorption at 800 nm as marker during the acute toxicity test.** C (control); 1.5 mg/l 2-MNQ concentration; 3 mg/l 2-MNQ concentration; 6 mg/l 2-MNQ concentration Diff., C (control); 1.5 mg/l 2-MNQ concentration; 3 mg/l 2-MNQ concentration; 6 mg/l 2-MNQ; concentration Diff., test statistics.

|  | (I) Treatment | (J) Treatment | Mean Difference (I-J) | Std. Error | Sig. | 95% Confidence Interval | |
| --- | --- | --- | --- | --- | --- | --- | --- |
|  |  |  |  |  |  | Lower Bound | Upper Bound |
| Dunnett T3 | Control | 1.5 mg/l | .0503^*^ | .01598 | **.047** | .0006 | .0999 |
|  |  | 3 mg/l | .0617^*^ | .01442 | **.009** | .0156 | .1077 |
|  |  | 6 mg/l | .1075^*^ | .01409 | **.000** | .0620 | .1530 |
|  | 1.5 mg/l | Control | -.0503^*^ | .01598 | **.047** | -.0999 | -.0006 |
|  |  | 3 mg/l | .0114 | .01284 | .929 | -.0289 | .0517 |
|  |  | 6 mg/l | .0573^*^ | .01247 | **.005** | .0178 | .0967 |
|  | 3 mg/l | Control | -.0617^*^ | .01442 | **.009** | -.1077 | -.0156 |
|  |  | 1.5 mg/l | -.0114 | .01284 | .929 | -.0517 | .0289 |
|  |  | 6 mg/l | .0458^*^ | .01039 | **.005** | .0136 | .0780 |
|  | 6 mg/l | Control | -.1075^*^ | .01409 | **.000** | -.1530 | -.0620 |
|  |  | 1.5 mg/l | -.0573^*^ | .01247 | **.005** | -.0967 | -.0178 |
|  |  | 3 mg/l | -.0458^*^ | .01039 | **.005** | -.0780 | -.0136 |

**Table S5: Post hoc tests for ROS formation in A. obliquus:** C (control); 0.075mg/l075 mg/l 2-MNQ concentration; 0.7575 mg/l 2-MNQ concentration; 1.5 mg/l 2-MNQ concentration Diff., test statistics.

| (I)Treatment | (J)Treatment | Mean Difference (I-J) | Std. Error | Sig. | 95% Confidence Interval | |
| --- | --- | --- | --- | --- | --- | --- |
|  |  |  |  |  | Lower Bound | Upper Bound |
| Control | DMSO | 12.1333 | 8.39783 | .601 | -11.2731 | 35.5398 |
|  | 0.075 mg/l 2-MNQ | -28.5889^*^ | 8.39783 | **.009** | -51.9953 | -5.1825 |
|  | 0.75 mg/l 2-MNQ | -532.5667^*^ | 8.39783 | **.000** | -555.9731 | -509.1602 |
|  | 1.5 mg/l 2-MNQ | -1486.1667^*^ | 8.39783 | **.000** | -1509.5731 | -1462.7602 |
| DMSO | Control | -12.1333 | 8.39783 | .601 | -35.5398 | 11.2731 |
|  | 0.075 mg/l 2-MNQ | -40.7222^*^ | 8.39783 | **.000** | -64.1286 | -17.3158 |
|  | 0.75 mg/l 2-MNQ | -544.7000^*^ | 8.39783 | **.000** | -568.1064 | -521.2936 |
|  | 1.5 mg/l 2-MNQ | -1498.3000^*^ | 8.39783 | **.000** | -1521.7064 | -1474.8936 |
| 0.075 mg/l 2-MNQ | Control | 28.5889^*^ | 8.39783 | **.009** | 5.1825 | 51.9953 |
|  | DMSO | 40.7222^*^ | 8.39783 | **.000** | 17.3158 | 64.1286 |
|  | 0.75 mg/l 2-MNQ | -503.9778^*^ | 8.39783 | **.000** | -527.3842 | -480.5714 |
|  | 1.5 mg/l 2-MNQ | -1457.5778^*^ | 8.39783 | **.000** | -1480.9842 | -1434.1714 |
| 0.75 mg/l 2-MNQ | Control | 532.5667^*^ | 8.39783 | **.000** | 509.1602 | 555.9731 |
|  | DMSO | 544.7000^*^ | 8.39783 | **.000** | 521.2936 | 568.1064 |
|  | 0.075 mg/l 2-MNQ | 503.9778^*^ | 8.39783 | **.000** | 480.5714 | 527.3842 |
|  | 1.5 mg/l 2-MNQ | -953.6000^*^ | 8.39783 | **.000** | -977.0064 | -930.1936 |
| 1.5 mg/l 2-MNQ | Control | 1486.1667^*^ | 8.39783 | **.000** | 1462.7602 | 1509.5731 |
|  | DMSO | 1498.3000^*^ | 8.39783 | **.000** | 1474.8936 | 1521.7064 |
|  | 0.075 mg/l 2-MNQ | 1457.5778^*^ | 8.39783 | **.000** | 1434.1714 | 1480.9842 |
|  | 0.75 mg/l 2-MNQ | 953.6000^*^ | 8.39783 | **.000** | 930.1936 | 977.0064 |

**Table S6: D. magna clones and sampling sites for the mesocosm study.**

| *D. magna* Clone | Sampling site |
| --- | --- |
| K17B | Fishpond; Ismaning, Germany 1997 |
| K17E | Fishpond; Ismaning, Germany 1997 |
| K17G | Fishpond; Ismaning, Germany 1997 |
| K34C | Former fishpond; Ismaning, Germany 1998 |
| K34H | Former fishpond; Ismaning, Germany 1998 |
| K34J | Former fishpond; Ismaning, Germany 1998 |
| K34K | Former fishpond; Ismaning, Germany 1998 |
| K34M | Former fishpond; Ismaning, Germany 1998 |
| K34Q | Former fishpond; Ismaning, Germany 1998 |
| K34X | Former fishpond; Ismaning, Germany 2014 |
| K34Y | Former fishpond; Ismaning, Germany 2014 |
| K36A | Former fishpond; Ismaning, Germany 2014 |
| K36B | Former fishpond; Ismaning, Germany 2014 |

**Table S7: Substances and their concentrations for the enriched tap water mesocosm medium per litre.**

| **Substance** | **Concentration** |
| --- | --- |
| CaCl_2_ | 1 mL |
| SeO_2_ | 10 µL |
| SeaSalt | 100 g |
| SMB-Buffer | 75 mL |

**Table S8 Recipe for SMB-Buffer.**

| **Stock solution** | **Substance** | **Concentration** |
| --- | --- | --- |
| Stock solution 1  5 ml | NaCl | 17.53 g/l |
|  | KCl | 0.74 g/l |
|  | CaCl_2_ * 2H_2_O | 11.76 g/l |
|  | MgCl_2_ * 6H_2_O | 2.03 g/l |
|  | MgSO_4_ * 7H_2_O | 2.46 g/l |
| Stock solution 2  10 ml | NaH_2_PO_4_ * H_2_O | 27.60 g/l |
|  | NaH_2_PO_4_ * 2H_2_O | 35.60 g/l |
|  | Aqua bidest | 1000 ml |
